# Velocity-Selected Rotational State Distributions of Nitric Oxide Scattered off Graphene Revealed by Surface-Velocity Map Imaging

**DOI:** 10.1021/acs.jpca.2c06196

**Published:** 2023-01-26

**Authors:** Thomas Greenwood, Huda AlSalem, Sven P. K. Koehler

**Affiliations:** † Department of Natural Sciences, Manchester Metropolitan University, ManchesterM1 5GD, U.K.; ‡ Department of Chemistry, College of Science, Princess Nourah bint Abdulrahman University, P.O. Box 84428, Riyadh11671, Saudi Arabia; § Institut für Verfahrenstechnik, Energietechnik und Klimaschutz, Hochschule Hannover, Ricklinger Stadtweg 120, 30459Hannover, Germany

## Abstract

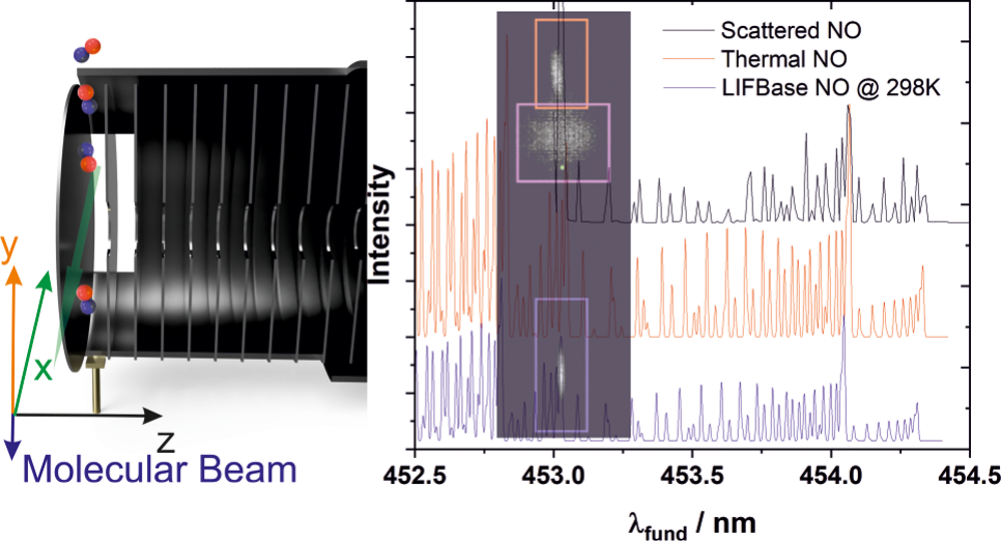

We report velocity-dependent internal energy distributions of nitric oxide molecules, NO, scattered off graphene supported on gold to further explore the dynamics of the collision process between NO radicals and graphene. These experiments were performed by directing a molecular beam of NO onto graphene in a surface-velocity map imaging setup, which allowed us to record internal energy distributions of the NO radicals as a function of their velocity. We do not observe bond formation but (1) major contributions from direct inelastic scattering and (2) a smaller trapping–desorption component where some physisorbed NO molecules have residence times on the order of microseconds. This is in agreement with our classical molecular dynamics simulations which also observe a small proportion of two- and multi-bounce collisions events but likewise a small proportion of NO radicals trapped at the surface for the entire length of the molecular dynamics simulations (a few picoseconds). Despite a collision energy of 0.31 eV, which would be sufficient to populate NO(*v* = 1), we do not detect vibrationally excited nitric oxide.

## 1 Introduction

Internal state distributions of radicals after reactions (where reactions include both chemical reactions and pure collision processes) allow us a glimpse of the detailed dynamics of such processes.^1,2^ This is because knowing the degrees of freedom into which some of the available energy is channeled enables us to learn about the flow of energy during the entire reaction and to draw conclusions from that information with regard to the actual dynamics of the process.^3^ Most famously, the Polanyi rules (broadly speaking) allow us to determine the position of the transition state of a chemical reaction along the reaction coordinate based on the vibrational state distribution of the products.^4^ Equally, the rotational state distributions can yield information about the geometry of a transition state,^5,6^ and phenomena such as rainbow scattering can provide insight into oscillatory behavior during reactions.^7,8^

One of the collision processes that has garnered much attention in the past few years is between atoms or molecules and graphene, i.e., the scattering off a graphene surface. The modification of graphene by chemical reactions on its surface to form covalent bonds is technologically important as such functionalizations can introduce a tunable band gap (pristine graphene is a zero band gap material),^9,10^ and scattering studies of graphene can reveal the fundamentals of the collision process and the potential bond-formation process on the graphene surface. While much of the experimental and computational work focused on translational energy and angular distributions of the scattered particles (the only possible degree of freedom for atomic collisions),^11−16^ Juaristi and co-workers,^17^ Rutigliano and Pirani,^18^ and Hase and co-workers^19^ also explored the rotational state distribution of O_2_ and N_2_ scattered off graphite. Hase and co-workers found that only a small fraction of the available energy is channeled into rotations but none into vibrations. Our group has previously investigated the translational energy distribution of NO after scattering off graphene supported on gold and detected a significant loss of ∼80% of the molecules’ kinetic energy and a surprisingly narrow angular distribution.^20^ In addition to this, we can also learn from state-resolved scattering studies off graphite.^8,21^ Walther and co-workers found that cold surfaces could lead to a cooling of the rotational temperature of the NO radicals in a rotationally hot molecular beam after collision with the graphite;^22^ however, a cold molecular beam of NO tends to result in a hotter rotational temperature for those NO radicals that are quasi-specularly scattered and an even hotter rotational distribution close to the surface temperature for the diffusely scattered NO molecules, i.e., the trapping–desorption component.^23^ Higher surface temperatures lead to hotter rotational temperatures of the specularly scattered NO.^24^ The same group found that at cryogenic graphite temperatures, the rotational temperature of the scattered NO is almost constant, and the group concluded that the formation of a short-lived collision complex which unimolecularly decomposes is responsible for this almost constant rotational distribution.^25^ Nyman et al. also found a rotational temperature of the NO after collision with graphite colder than the surface temperature (rotational cooling) in their modeling studies and even rotational rainbows at higher surface temperatures.^26^

We hence set out to record rotational state distributions of nitric oxide radicals after collisions with graphene in our surface-velocity map imaging (VMI) setup.^27,28^ VMI (typically applied to gas-phase dynamics) has recently been applied more and more to study surface dynamics,^29−33^ allowing one to derive speed and angular distributions of the scattered products. A resonance-enhanced multiphoton ionization (REMPI) scheme is frequently applied in VMI studies, guaranteeing state-selectivity. The beauty of combining VMI with REMPI in these surface scattering studies here is due to the fact that while REMPI spectra traditionally integrate over the entire ion yield, we can now define regions of interest (ROI) in the VM images (which record the velocity information) and only sum over the ion yield in those ROIs, corresponding in our case to NO radicals flying with a certain speed in a certain direction.^34^ Since we measured the velocity distributions of NO after scattering off graphene already,^20^ we now investigate the rotational state distributions of the various components that make up the scattered cloud (i.e., inelastically scattered and trapping-desorbed NO) in the same experiment to derive information about the collision dynamics of NO radicals with graphene.

## 2 Methodology

Our surface-VMI apparatus has been described previously,^20^ but specifics to the measurement of velocity-selected rotational state distributions are briefly described here. The skimmed (Beam Dynamics, 0.5 mm) molecular beam of ∼2% NO in He (a General Valve series 9 valve is used; the fwhm of the kinetic energy distribution is ∼0.08 eV; see Figure S1) is expanded into the main chamber housing the graphene surface (at room temperature) at a base pressure of 5 × 10^–9^ Torr, increasing to 3 × 10^–8^ Torr when the beam is on. The NO molecules fly toward the graphene along the surface normal and are intersected by the REMPI laser at right angle in the center of the VMI chamber twice, both on the way toward the surface and after the scattering event. Nitric oxide molecules are ionized in a (1 + 1) REMPI scheme (via the A^2^Σ state) using the frequency-doubled output of a Radiant Dyes NarrowScan laser running on coumarin 450, yielding pulse energies of ∼0.8 mJ at around 227 nm with a resolution of around 0.08 cm^–1^. The doubled dye laser output is unfocused due to the ease with which NO can be ionized. The ionized NO particles are then accelerated toward the position-sensitive detector (in a direction normal to the plane spanned by the molecular beam and the laser; see Figure S3) where the images are recorded by a NET GmbH CMOS camera. The time at which the laser fires in relation to the opening of the molecular beam is adjusted via an SRS DG645 delay generator. This adjustment of the timing is necessary to select for events in the scattering process which are separated in time such as (1) the molecular beam on the way down to the surface or (2) once it has scattered which is usually 100–300 μs later. Due to the opening time of the molecular beam (∼300 μs), the signal on the detector can be a combination of molecular beam and scattered molecules, but crucially, NO molecules in the molecular beam (which are flying “downward” in the lab frame) appear in the lower half of the velocity-mapped image, i.e., below our zero velocity pixel, while scattered NO molecules with an upward velocity component in our laboratory frame appear in the upper half of the detector. We can thus differentiate the various components of the beam and the scattering event not only by varying the delay time between the molecular beam and the REMPI laser but in a much better way by observing certain ROIs on the detector. We hence extract rotational distributions of scattered NO molecules for (1) a fast and narrow spot presumably due to direct inelastic scattering and (2) a much weaker component likely due to trapping–desorption which appears as a broad and slower diffuse cloud in the images, and we thus integrate over the ion signals on the imaging detector for the various components separately by concentrating on the ROI relevant to each component.

The accompanying classical molecular dynamics simulations have been described previously.^35^ The gold substrate was formed of a 6 × 6 × 6 array of gold atoms, and 98 carbon atoms were positioned in a hexagonal 2D network in the *x*–*y* plane on top of the gold substrate. Periodic boundary conditions were applied along the *x*–*y* plane but with no periodicity in the *z* dimension. We stress that these are purely classical calculations which do not account for the quantum behavior of molecular motions.

## 3 Results and Discussion

**Figure 1:**
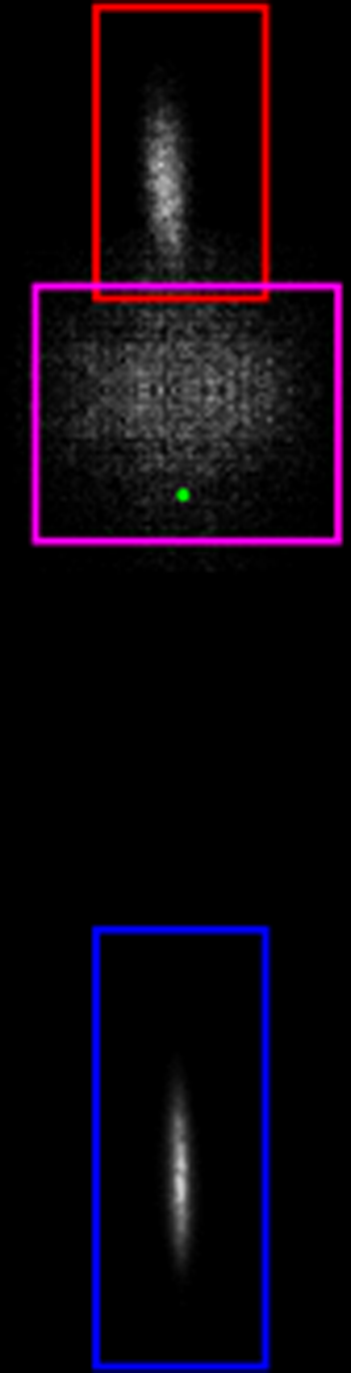
Images of the various NO components along with their respective regions of interest: blue for NO in the molecular beam at a relative time (*t*_rel_) of 0 μs, red for scattered NO (*t*_rel_ + 100 μs), pink for the trapping component (*t*_rel_ + 400 μs), and the green dot indicating our center spot relating to zero overall velocity in the *x* and *y* dimensions.

Before delving into the rotational spectra, we first briefly discuss the various NO species we investigated. As discussed in the Methodology section, the various rotational spectra were collected by integrating the ion signal only over certain ROI on the detector. These regions are shown in Figure 1, which itself is a composite image consisting of individual images recorded at different delay times between the molecular beam and the REMPI laser, purely for the benefit of highlighting the different components.

One can observe the signal from the molecular beam itself (within the blue rectangle in Figure 1), the directly scattered component (red), and a slow (but crucially upward in the lab frame, pink) component. This slower component, which we assign to a trapping desorbing mechanism, is much weaker than the scattered component, and we in fact failed to observe it in our previous work.^20^ While it appears as if some components may overlap in the images, most notably the signal for the direct inelastic scattering and the slower trapping component, varying the delay time between the molecular beam and the REMPI laser allows us to separate the integrated signals. Relative to the molecular beam itself, which was recorded at its peak in the time-of-flight profile, the directly scattered NO signal was recorded 100 μs after the molecular beam in order to image the “same” NO molecules that were imaged in the beam initially, and the trapping–desorption component was recorded a further 300 μs after the directly scattered NO.

The trapping–desorption component is rather wide such that not all ions detected follow true VMI conditions, and due to the long image acquisition time, noise and background ions are appearing.

Table 1 shows the average velocities of those events; not shown in the table is the thermal gas-phase background NO (see section 4 of the Supporting Information), which has been discussed previously,^20^ but briefly, nitric oxide gas was admitted into the chamber through a leak valve at a temperature of 298 K. Imaging those thermal background molecules yields a two-dimensional Maxwell–Boltzmann distribution on the detector whose center is taken to be the zero-velocity center of our actual images, i.e., the green dot in Figure 1. A composite image of all components including the thermal background spot is shown in Figure S7 of the Supporting Information, highlighting the difference in position between the thermal background image and the trapping–desorption components. The three components (molecular beam, direct scatter, trapping desorption) were velocity-analyzed separately and with respect to the center spot of zero velocity using a conversion factor of 5.1 m s^–1^/pixel, fitted to eq 1,
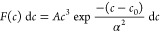
1where *A* is a scaling factor and α is related to the width of the distribution,^36^ and errors are standard errors.

**Table 1:** Various NO Species Detected in Our Experiment with Their Respective Speeds at Peak Delay Times^a^

event	beam	scatter	trapping–desorption
*v*, m s^–1^	1428 ± 114	638 ± 108	184 ± 161

a The beam is naturally traveling in opposite direction to the scatter and trapping–desorption components.

The average translational energy of the Maxwell–Boltzmann distribution of particles originating from a flat surface is 2*kT* (equivalent to ∼575 m s^–1^ for nitric oxide desorbing from a 298 K surface); thus the directly scattered component is despite the large energy loss noticeable faster, while the trapping–desorption component is slower.

**Figure 2:**
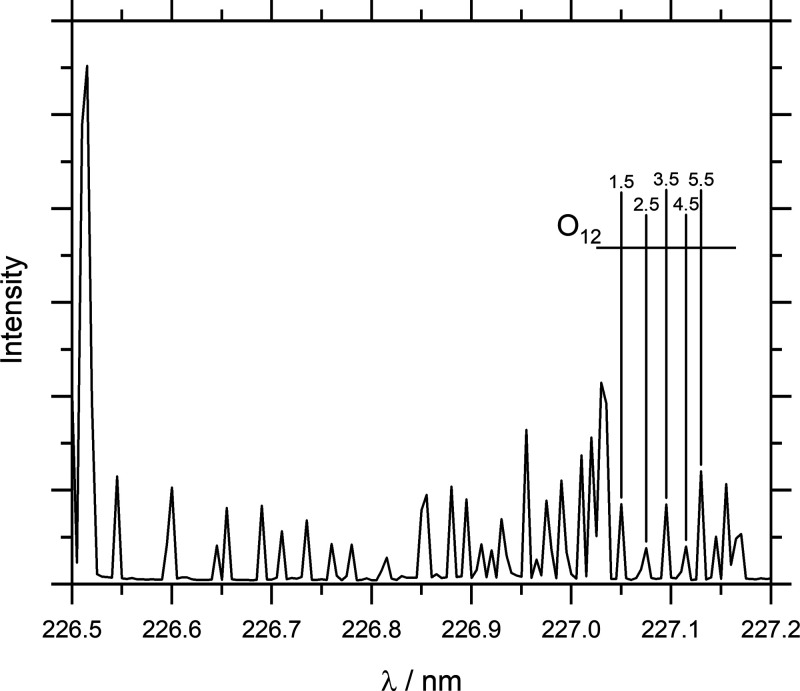
Rotational spectrum of the scattered NO with the relevant lines of the O_12_ branch highlighted.

Rotationally resolved REMPI spectra were recorded by scanning the laser over the desired wavelength range (at the appropriate delay time) while using the MCP detector in imaging mode and by integrating the ion signal in each of the three ROIs separately for each wavelength. The resulting spectra are then converted to rotational state populations using a relative calibration scheme by means of comparison with a thermal background spectrum in LIFBase as a reference.^37^ Only the O_12_ and P_12_ branches were recorded to avoid overlapping lines, as in particular the first 20 transitions of the O_12_ branch do not overlap with any other lines. A raw REMPI spectrum of scattered NO is shown in Figure 2, and a composite of a scattered, thermal background and LIFBase simulation spectra are shown in Figure S8 of the Supporting Information.

**Figure 3:**
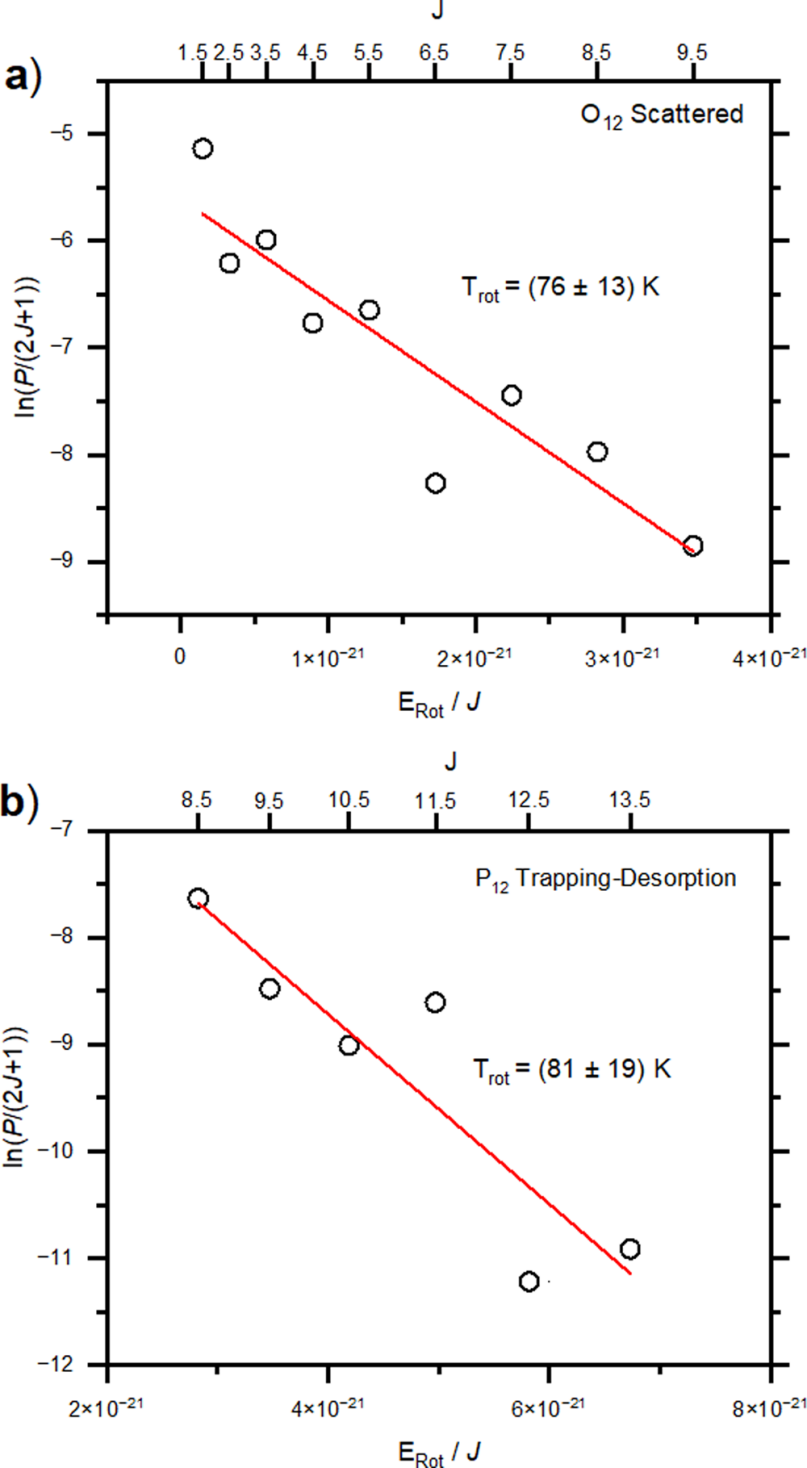
(a) Boltzmann plot of O_12_ branch of the scattered NO. (b) P_12_ branch of the trapping desorption component.

Once the populations of the rotational energy levels were assigned, Boltzmann plots were created for each branch. Example plots from the O_12_ branch of the scattered NO and the P_12_ branch of the trapping desorption component are shown in Figure 3.

Rotational temperatures for each component were derived from the gradients of these Boltzmann plots. While there is no reason for either rotational distribution here to follow a Boltzmann distribution, the assignment of a single rotational temperature eases comparison between the components. The temperatures of the various NO components are shown in Table 2.

**Table 2:** Rotational Temperatures of the Various NO Events Derived from the Linear Fits to the Boltzmann Plots in Figure 3^a^

event	beam	scatter	trapping–desorption
*T*_rot_, K	64 ± 11	76 ± 11	81 ± 19

a Errors are standard errors.

As is clear from the velocities derived from the images and now the rotational temperatures, there are two channels from the scattering of NO, the directly scattered component and a trapping–desorption component. Both components gain some rotational energy, which is modest, but despite the errors discernible. The directly scattered component is likely only undergoing a single collision with the surface and expectedly does not gain much rotational energy. However, even the trapping–desorption component (despite losing a significant amount of translational energy) does not gain much more rotational energy as compared to the directly scattered component. It appears as if the NO molecules transfer much of their translational energy to the graphene substrate in just a few collisions, but it is possible that only those with a cold(er) rotational temperature are scattered back, while those that are (partly) thermalizing may become trapped, leading to the relatively cold rotational state distribution of the trapping–desorption component. Our previous MD simulations for NO scattering off graphene can shed some light onto that,^35^ though they do not compare quantitatively and do not reproduce the two well-separated scattered components observed here (directly scattered and trapping–desorption). In the simulations, the majority of NO scatters off graphene directly, undergoing only a single collision, but there is a small fraction of NO molecules that interact with the surface for only a couple of picoseconds before desorbing again (though these do not show up as a separate component in our time-of-flight profiles), presumably undergoing too few collisions to rotationally thermalize.

Directly scattered and diffuse spots for scattered NO have also been recorded after interactions with graphite.^23^ A slower diffuse component was observed which was likely to be due to temporary, i.e., nonequilibrium, trapping, similar to our experimental results.

This behavior of incomplete thermalization of NO has also been identified before in the scattering off graphite,^24,38^ where NO only accommodates to the surface temperature up to 170 K, above which the surface temperature and the rotational temperature deviate. However, a direct comparison to our experiments is not entirely justified due to the different properties of graphene vs graphite, of course, but mainly due to the different incidence angles.

A further notable difference, however, is that the rotational temperatures of both components in our study here on graphene are rotationally cooler than previous studies of scattering of NO off graphite at around room temperature, and our values align better with those rotational temperatures measured at graphite temperatures of around 80 K as reported by Häger et al.^25^ In the case of NO scattered off graphite at 80 K, the resulting rotational temperatures were 88 and 90 K for the directly scattered and trapped NO, respectively, similar to our results, though our experiments were performed at a surface temperature of 298 K; however, incidence angles are not the same in these two studies. It is worth highlighting, though, the fact that in the studies by Häger et al. as well as in our studies here, the rotational temperatures of the two components do not differ much.

While experimental studies of NO scattering off silver have led to rotational rainbows,^39^ and we have found evidence for those in our MD simulations, no rotational rainbows were observed in this experimental study here or in other experimental studies of NO on graphite.^26^

## 4 Conclusions

A molecular beam of nitric oxide molecules was scattered off graphene supported on gold, and the velocities and internal energy distributions of the scattered NO molecules were probed using a surface-velocity map imaging setup. No bond formation was observed at the graphene surface, and despite sufficient collision energies of 0.31 eV, no vibrationally excited NO molecules were observed. Two components of scattered NO were observed, namely, (1) a directly scattered component and (2) a trapping–desorption component which had lost a significant proportion of its initial translational energy. This compares at least qualitatively with our previous MD simulations in which most NO molecules collide with the graphene surface only once, but a small fraction undergoes multiple collisions, though in our MD work, this latter component could not be separated clearly from the direct component based on time-of-flight profiles.

Both the direct and the trapping–desorption components gain some rotational energy but are remarkably similar in their rotational temperatures, with the trapping–desorption component only marginally hotter but far away from having reached thermal equilibrium with the surface.

This fairly unusual behavior appears typical for graphene with its perfectly flat structure, but since we were limited to incidence angles along the surface normal and room temperature surfaces in these studies, we intend to perform further studies to shed light onto this behavior in graphene scattering.

## Supporting Information Available

Experimental setup, raw data, molecular beam characterization, calibration of VMI setup and beam positions, and the spectroscopy of nitric oxide (PDF)
