# Supplementary material for: Velocity-Selected Rotational State Distributions of Nitric Oxide Scattered off Graphene Revealed by Surface-Velocity Map Imaging
Source: J Phys Chem A. 2023 Jan 26;127(5):1124–9. doi: 10.1021/acs.jpca.2c06196 (PMC9923741; doi:10.1021/acs.jpca.2c06196)
Supplement: Supplementary file 1 — jp2c06196_si_001.pdf [file jp2c06196_si_001.pdf]

SUPPORTING INFORMATION

Velocity-selected Rotational State Distributions of Nitric Oxide Scattered off Graphene Revealed by Surface-Velocity Map Imaging

Thomas Greenwood,[1] *Huda AlSalem*,[2] *Sven P. K. Koehler**[1,3]

[1] Department of Natural Sciences, Manchester Metropolitan University, M1 5GD, UK

[2] Department of Chemistry, College of Science, Princess Nourah bint Abdulrahman University, P.O. Box 84428, Riyadh 11671, Saudi Arabia

[3] Hochschule Hannover, Institut für Verfahrenstechnik, Energietechnik und Klimaschutz, Ricklinger Stadtweg 120, 30459 Hannover, Germany.

*Corresponding author.

Email: sven.koehler@hs-hannover.de

Hochschule Hannover

Institut für Verfahrenstechnik, Energietechnik und Klimaschutz

Ricklinger Stadtweg 120

30459 Hannover

Germany

1. Energy Spread of Molecular Beam

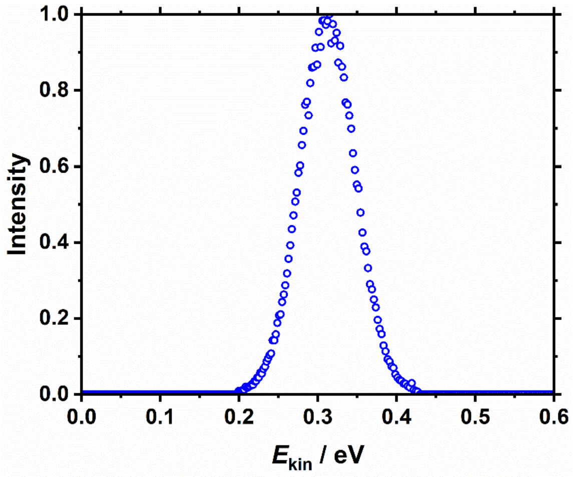

Figure S1: Kinetic energy distribution of the incoming beam of 2% nitric oxide, NO, in He.

2. Overall time-of-flight distributions

The time-of-flight distributions recorded as a signal on the oscilloscope integrating over all regions of the detector (in black), and the same distributions but recorded for different regions of interest showing the molecular beam (red), the directly scattered component (blue), and the trapping-desorption component (green).

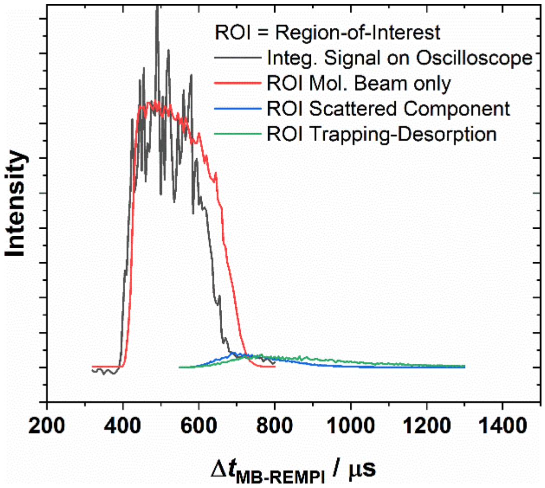

Figure S2: Overall time-of-flight distribution measured on the scope (in black) and distributions measured using Regions of Interest (ROI)

At a repetition rate of 10 Hz and hence a duty cycle of 100 ms or 100,000 μs, one can see that the scattered signal all but disappears around 1300 after the molecular beam fires, and contributions to our signal from background NO is negligible.

3. Experimental Setup

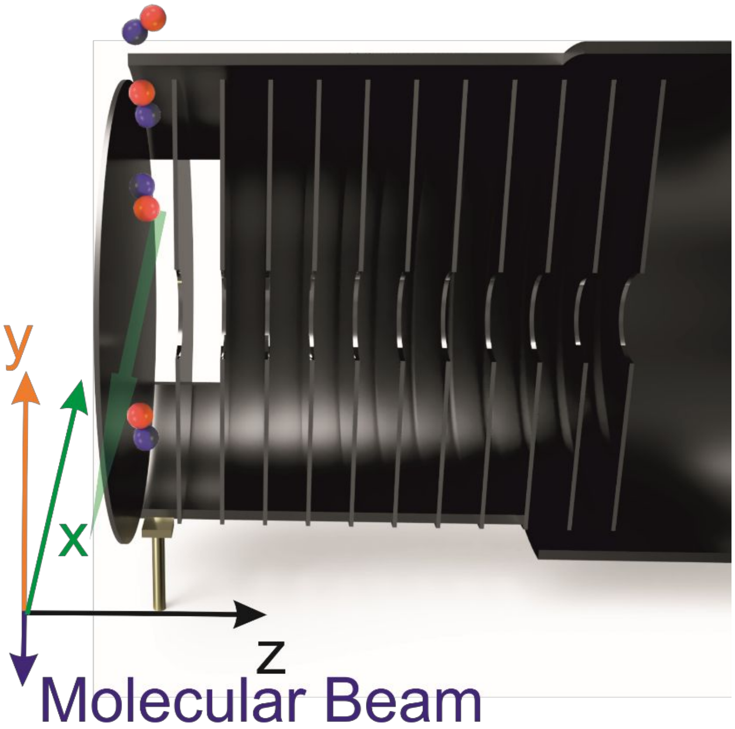

Figure S3: Relative orientation of the laser (*x*, into the plane of the paper), the molecular beam (*y*, downwards), and the time-of-flight direction towards the multi-channel plate detector (*z*).

The molecular beam is created using a General Valve Series 9 valve with an opening time of around 300 μs. The expansion passes through a skimmer (Beam Dynamics, 0.5 mm) placed 30 mm

away from the valve. The graphene surface is mounted 320 mm away from the valve such that a beam of ca. 5.5 mm diameter is impinging on the graphene surface.

The sample holder is a simple stainless-steel cylinder of 5 mm diameter on which the graphene sample rests. The sample consists of a 10 × 10 mm^2 quartz wafer covered with a thin layer of evaporated gold on to which the graphene adheres (after having been transferred using PMMA). The dimensions of the graphene are barely smaller than the wafer itself, hence the 5.5 mm diameter molecular beam impinges mostly on graphene indeed.

The ~227 nm laser beam is unfocussed and around 7 mm in diameter, though can be cut down using an iris. Due to the equal ionisation efficiency along the laser path (unfocussed beam), we can ionise any NO molecule within the laser beam, but only those within 10 mm either side of the center axis will be able to be accelerated to the detector, allowing a detection angle of 23°.

We have previously shown that VMI conditions prevail in our spectrometer up to 6-7 mm either side of the centre axis,[1] i.e. we have a small proportion of NO molecules here that are not focussed truly under VMI conditions, but only the small fraction further away from the surface normal.

4. Calibration

Figure S4 shows an image of thermal nitric oxide at 298 K let into the chamber through a leak valve at 1 × 10^{-7} Torr. The same image is shown in 3D as Figure S5, left image. A fit of this thermal background image to a 2-dimensional Maxwell-Boltzmann distribution is shown on the right of Figure S5, and allows us to determine the zero velocity pixel on our detector shown as a green dot in Figure S4.

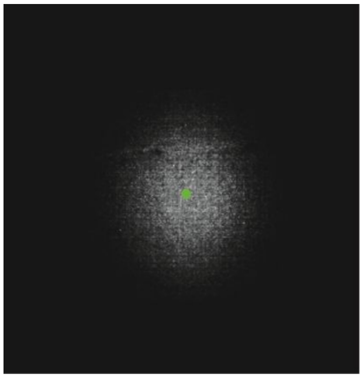

Figure S4: Thermal background image of nitric oxide at 298 K with zero velocity spot indicated.

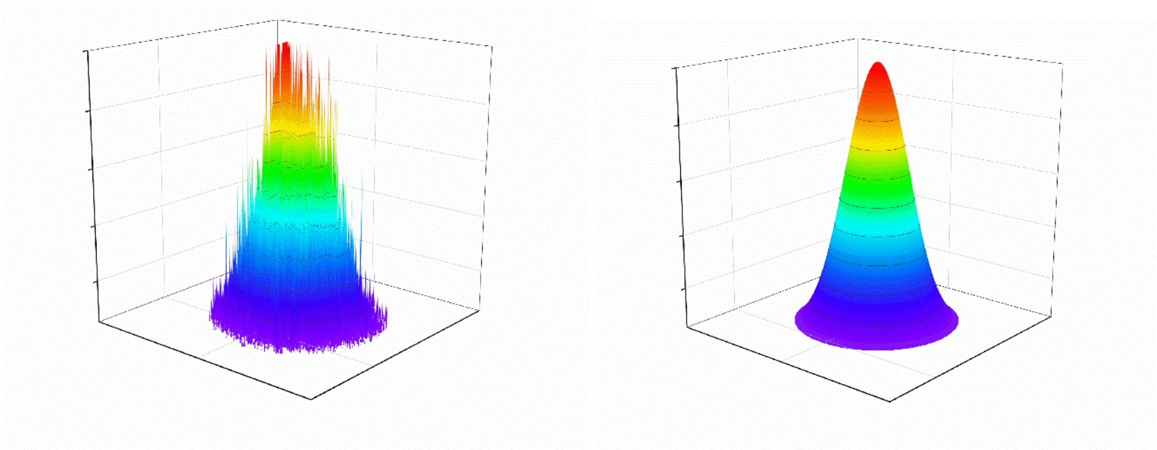

Figure S5: Raw thermal background image of nitric oxide let into the vacuum chamber through leak valve at 1 × 10^{-7} Torr (left), and fit of a 2-dimensional Maxwell-Boltzmann distribution to the thermal background spot (right) to determine zero velocity pixel shown in green in Figure S4.

5. Nitric Oxide Energy Level Diagram

Figure S6 shows an energy level diagram of nitric oxide for the vibrational ground states of the electronic ground states X $^2\Pi_{1/2}$ and X $^2\Pi_{3/2}$, and the first excited A $^2\Sigma^+$ state, including one of the probed lines, namely the $O_{12}(3.5)$ transition.

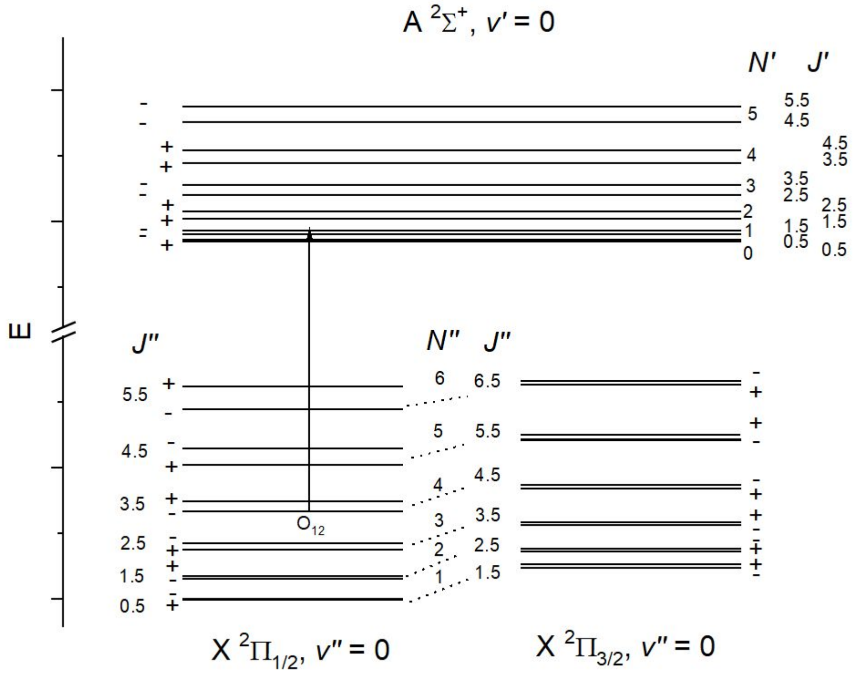

Figure S6: Energy level diagram of nitric oxide including the probed $O_{12}(3.5)$ line.

6. Image Positions of Molecular Beam, Direct Scatter and Trapping-Desorption Component in Relation to the Thermal Background Image

Figure S7 shows a composite image showing the positions of the molecular beam, directly scattered image and trapping-desorption component in relation to the thermal background image (in red). We note that the images were recorded at different delay times between the molecular beam and the REMPI laser (or even under different conditions in the case of the red background image), such that we can differentiate the components both by their timing (through some components overlap) as well as by their position on the detector. While the camera might move in the horizontal plane slightly, its vertical alignment is far more robust, and the trapping-desorption spot can always be seen to have an overall upward velocity component. Furthermore, the trapping-desorption spot decreases in intensity as a function of time.

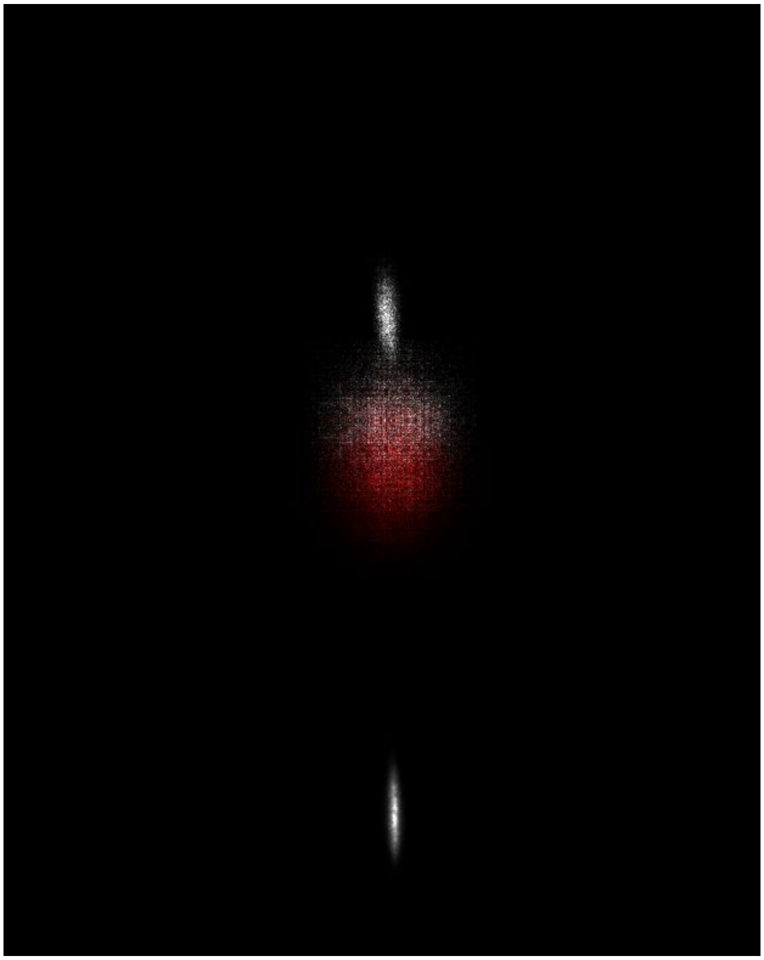

Figure S7: Composite image of the molecular beam (white bottom), directly scattered (white top) and trapping-desorption component (fuzzy white in the middle) in relation to the thermal background image in red.

7. Rotational Spectra

Figure S8 shows a rotational spectrum of the scattered NO (at the fundamental of the laser, not the doubled), a rotational spectrum of the thermal background NO, and a LIFBase simulation of 298 K thermal NO, demonstrating the cold rotational temperature of the scattered component. The latter two are not identical due to e.g. saturation effects, .[2]

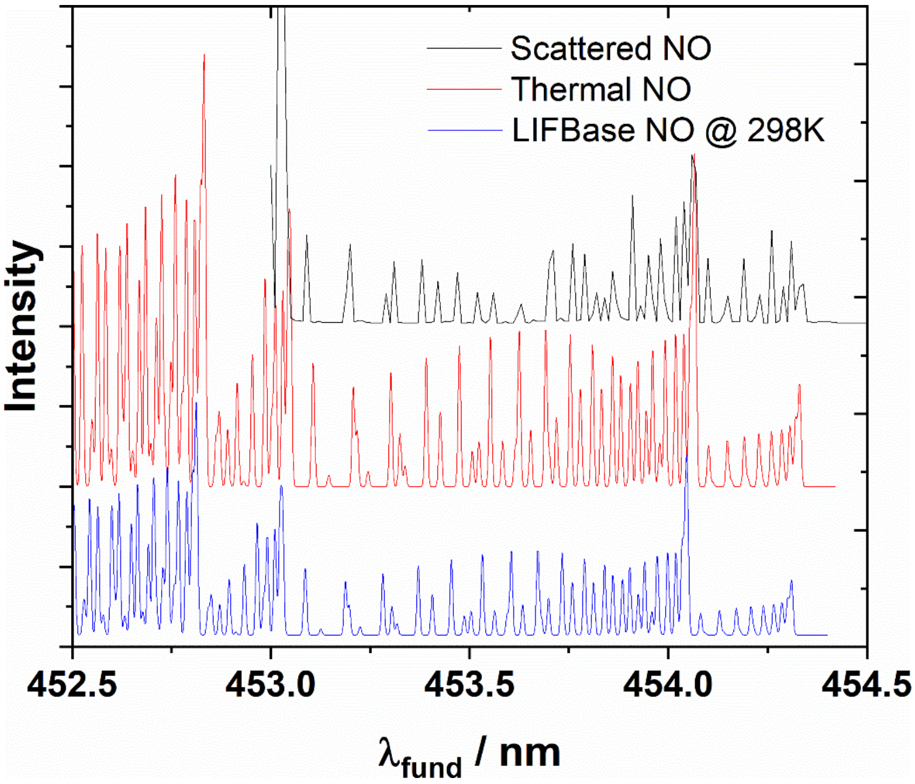

Figure S8: Rotational spectra of scattered NO (black), thermal background NO (red), and a LIFBase simulation of 298 K (blue). The LIFBase spectrum was shifted by 0.2 nm to coincide with the experimental spectra as the laser wavelength was not calibrated.

[1] Mike Reid and Sven P. K. Koehler, Validation of velocity map imaging conditions over larger areas, *Rev. Sci. Instrum.,* 2013, **84**, 044101--0441016.

[2] Sven P. K. Köhler, Mhairi Allan, Hailey Kelso, David A. Henderson, Kenneth G. McKendrick, The effects of surface temperature on the gas-liquid interfacial reaction dynamics of O(3P) + squalane, *J. Chem. Phys.* 2005, **122**, 024712-0247129.
